# Transitional Care in Rheumatology: a Review of the Literature from the Past 5 Years

**DOI:** 10.1007/s11926-019-0855-4

**Published:** 2019-09-06

**Authors:** Janet E. McDonagh, Albert Farre

**Affiliations:** 1 Versus Arthritis Centre for Epidemiology, Centre for MSK Research, University of Manchester and NIHR Biomedical Research Centre, Manchester University Hospital NHS Trust, Manchester, UK; 2 School of Nursing and Health Sciences, University of Dundee, Dundee, UK

**Keywords:** Transitional care, Rheumatology, Chronic illness, Adolescents, Young adults, Review

## Abstract

**Purpose of Review:**

Effective transitional care for adolescents and young adults (AYA) with rheumatic musculoskeletal disease (RMD) is fundamental to rheumatology care provision. Here we review the recent evidence from the literature on transition in rheumatology and debate why universal implementation has yet to be recognised.

**Recent Findings:**

Evidence of need for transitional care continues to be reported. The triphasic nature of transitional care remains poorly recognised, and the third phase following transfer to adult rheumatology is particularly under-researched in spite of the recognition of the age-related trajectories of transition skill development during young adulthood. Several rheumatology-specific transitional care interventions have now been evaluated but the search for valid measures including outcome continues. Finally, the need to study transition at a health system level is increasingly recognised.

**Summary:**

Future research in this area should consider the developmental trajectories of AYA as well as the social-ecological model of transition readiness, which focuses on the interactions between AYA, caregivers and providers (and the systems they are part of) as these are the likely targets of any intervention to improve health transitions.

## Introduction

Effective transitional care for adolescents and young adults (AYA) is fundamental to rheumatology care provision, particularly given the continuing active disease and morbidity still observed in adulthood in many of the rheumatic musculoskeletal diseases (RMD) [1–3].

The concept of health transition first appeared in the medical literature over 35 years ago [4] and, a decade later, in the rheumatology literature [5]. Since then, the evidence base around transition has gradually grown [6] and its recognition has become more prominent on international rheumatology agendas, with the publication of specific guidance for professionals and policy makers [7, 8••].

After all these years, the concept of transition is often still primarily focused on the one-time event of transfer, in which AYA care is handed over from children-centred to adult-centred services. However, transitional care not only prepares AYA and their parents/caregivers for the differences between child and adult services and how to negotiate them but also supports the development of health literacy and self-management skills. This is a critical aspect of this life stage, where responsibility shifts from parents/caregivers to a shared responsibility and eventually to AYA themselves if they have capacity.

Clinically, transitional care involves addressing the evolving psychosocial and educational/vocational aspects of care alongside the traditional physical/medical aspects of care in a gradual, responsive, developmentally appropriate manner as AYA grow up. This also means that the planning and implementation of transitional care is relative to AYA development, rather than relative to a health system–defined age of transfer to adult care, thus requiring engagement from both child and adult services from around 11 to 25 years of age to support effective implementation of transitional care.

The aims of this paper are to critically review the recent evidence from the literature on transition in rheumatology, to debate why universal implementation has yet to be recognised and to propose some areas for future research agendas. Whilst studies on transition are typically carried out within one specific condition or group of conditions, such as RMD, a growing body of evidence suggests that condition-specific factors are only one factor in the context of broader common factors relating to the developmental trajectories of AYA [9] which mean that many barriers/enablers of transitional care are similar across different types/groups of conditions [10]. Therefore, this review will also consider recent evidence from non-rheumatology literature of relevance to transitional care in rheumatology, with a view to ensure shared learning with other conditions, areas and specialities.

## The Evidence of Need for Transitional Care

A recent survey of 115 rheumatology centres in 22 European countries (44% response rate) reported that 74% had transition services, although only 27% had a written transition policy and only a minority of teams had designated transition staff [11•]. Previous surveys have reported unmet training needs in rheumatology professionals, although there is no recent data specific to rheumatology, the last study being in 2004 [12]. However, recent reports of unmet training needs in both paediatrics [13, 14] and adult physicians [15] would suggest that there is still room for improvement.

In spite of the increasing awareness of the need for transitional care, AYA continue to report unmet needs in this area [16] and AYA with RMD have specifically called for more research on transition [17]. Many of these unmet needs are similar to those reported years previously [18] and echo the findings from studies of AYA with other long-term health conditions (LTHC) [19]. In a descriptive study involving 89 AYA (16–23 year olds) with a range of LTHC, 56% of which were rheumatic, nearly half reported never having had any discussion of transition and/or never seeing a healthcare provider independently for at least part of the visit [20].

Parents/caregivers are integral to transitional care and can become important facilitators, but in order to do so, they require clarification on their role in the transition process and support from service providers [21]. The importance of parents/caregivers was also echoed in the rheumatology literature, which found that the main indicator for transfer initiation, according to rheumatology professionals, was parental/caregiver perception of AYA readiness (reported by 62% of 138 surveyed professionals) [22]. Suris et al. [23] examined differences between parents of AYA with LTHC reporting an easy or difficult transfer to adult services. A third reported a difficult transfer. An easy transfer for parents was associated with feeling ready and considering that the coordination between teams was good [23].

## The Timing and Phases of Health Transition

Transitional care can be characterised as a three-stage process, involving the following: an initial lengthy phase of preparation, starting in early adolescence; a second shorter phase around the event of transfer, usually in late adolescence; and then a third phase of variable length following the event of transfer when AYA gradually engage with the new adult services.

Evidence on the optimal start of the first phase of the transition process had been reported in previous work in rheumatology, with evidence to support starting transitional care in early adolescence [24]. This finding has been echoed by more recent work in AYA with other LTHC [25] and embedded in national guidance across a number of countries, including the UK, USA and Canada [26–28]. One could argue that paediatric care becomes adolescent care around 11–12 years and hence is the developmentally appropriate time to start transitional care in view of the various other transitions which occur during adolescence (e.g. pubertal, social and vocational).

The second phase of the transition process, due to its focus around the event of transfer, tends to have fixed age criteria, influenced more by the healthcare system and less by the developmental status of the individual AYA. Jensen et al. [20] reported that only older age, but not transition readiness nor demographic variables, predicted transfer or time to transfer. A recent systematic review of reviews examining the impact of age of transfer on health and health service outcomes [29•] found moderate evidence that models of transition which transfer AYA in late adolescence or early adulthood can improve transition outcomes and patient satisfaction.

When it comes to the third and final phase of the transition process, there is less evidence available to support when the transition process ends. This often neglected and disproportionally under-researched phase of the transition process is of variable length and follows the event of transfer as the young person gradually engages with the new adult services. In a systematic review of systematic reviews, Hart et al. [30•] reported only 14 of 71 primary studies focused on this third phase. At this time, AYA are still developing (including brain development as well as other key psychosocial aspects of development) and there is great variation in maturity. This is reflected by the reported trajectories of transition skill acquisition, which extend well into the third decade. A study examining the longitudinal course of acquisition of healthcare transition skills of AYA with inflammatory bowel disease (IBD) [31] reported that whereas 50% of such skills were acquired by 12–14 years, the remainder were acquired in young adulthood (i.e. > 18 years) including skills in self-management, vocational development, health insurance, finding new healthcare providers and reproductive health. Another study of transition readiness skill acquisition in 16–25 year olds with IBD [32] reported mastery of only 9 of 20 transition readiness items, with key deficits in healthcare utilisation/self-advocacy (e.g. understanding insurance, making appointments) and self-management (e.g. filling/reordering prescriptions). Such data reinforces the need to consider this third phase of transition, both in practice and research, and the importance of ensuring the developmentally appropriateness of adult rheumatology services.

## Transitional Care Interventions

The first ever objective evaluation of an evidence-based, transitional care programme in rheumatology [33] was carried out over 15 years ago [24]. Transition readiness checklists (referred to as individualised transition plans) were developed as part of this programme and have since been adopted by others [34] and are specifically referenced in the European League Against Rheumatism (EULAR) guidance [8].

A systematic review and critical appraisal of existing transitional care programmes in rheumatology [35•] identified 8 transition programmes in 6 countries but found that within these programmes, there was variation in structures, staffing and processes with no standardised outcome or effectiveness measures.

Since the time of the latter review, three further evaluated programmes have been reported [36–38] (see Table 1), with only one of these [38] starting in early adolescence.

**Table 1:** Evaluated transitional care programmes in rheumatology reported in peer-reviewed journals since 2015

Reference	Programme	Country	Setting	Diseases	Age at enrolment (years)	Designated transition staff	Transition readiness assessment	Programme components	Shared care	Age of transfer	Evaluation	Results
Jensen et al. (2015) [36]	Social worker–centred paediatric rheumatology transition program	USA	Paediatric rheumatology clinic	Any RMD requiring transfer to adult rheumatology	Median 18 (15–26)	Social worker	Rheumatology-specific workbook including self-reflective questions Written transition goals reviewed at clinic visits	Face-to-face meetings with social worker at clinic visits and interim phone calls; facilitation of the adult appointment; phone call follow-up 6–8 months post transfer	No	When paediatric rheumatologists deemed appropriate	Satisfaction questionnaire Attendance at adult rheumatology clinic Compared with the control group who had not participated in the programme	81% satisfied 42% successful transition vs 23% controls (*p* = 0.002)
Stringer et al. (2015) [37]	Rheumatology transition clinic	Canada	Paediatric rheumatology clinic	Any RMD but majority JIA	2 years prior to completion of high school	None specific	No checklists used	3–6 combined MDT clinics per year with adult rheumatologist present	Yes	Aim to transfer when finished high school and their disease is quiescent 17–20 years	Questionnaire Follow-up rate post transfer	Overall satisfaction Variation in the adequacy of how transition issues were addressed High rate of follow-up post transfer
Walter et al. (2018) [38]	Clinical transition pathway for adolescents with juvenile-onset RMD	Netherlands	Adolescent clinic in adult rheumatology setting	RMD	12–14 years	None specific	Individualised transition plans (ITP) [33]	Clinical pathway Dedicated adolescent clinic	Yes	17–18 depending on the patient’s perceived skills, personal wishes in discussion with health professionals	Review of electronic patient records Frequency of dropout of care Use of ITPs Transfer experience: OYOF-TES [39] Self-efficacy scale OYOF-SES [40]	Reduced loss to follow-up High scores for satisfaction and self-efficacy

Reflecting the triphasic nature of transition discussed in the previous section, the literature does suggest that clinics for young adults (i.e. 16–25 year olds) are associated with better outcomes [41, 42]. However, a challenge for researchers in this area is the terminology surrounding transitional care and what is meant by a ‘transition clinic’, which can range from ad hoc clinics with the rest of care provided in ‘all-age clinics’ to clinics with multiple members of both the paediatric and adult teams present (sometimes in the same consultation room!). In a study of 23 clinics representing 14 specialties in a large paediatric hospital, 14 reported having a transition programme in place, but only 5 of these defined transition *holistically* (i.e. as addressing the medical, psychosocial and vocational aspects of care), and these 5 were found to have significantly greater levels of AYA satisfaction compared with those who did not define transition *holistically* [43].

Do all AYA need the same transition model? Is it possible to target the often-limited resources to those AYA who might need most support during this time? Hislop et al. [44] reported that when approaching transition, AYA adopted one of four broad interaction styles: ‘laid-back’, ‘anxious’, ‘seeking autonomy’ (being in control) or ‘socially oriented’ (welcoming support from and frequent discussions with family, friends and healthcare professionals). Such findings pose further questions: will the same transition model ever be appropriate for all these 4 interaction styles? Are these styles fixed or change over time as AYA grow up? Further research is thus eagerly awaited!

## Transition Measures

Assessments of both transition and transfer readiness are integral components of any clinical encounter with AYA with RMD, as recognised in international guidance [8••]. Such assessment promotes and facilitates opportunities for knowledge and skills training and helps track the individual AYA through the transition process. Assessment can also help identify AYA at risk of negative outcomes and enable professionals to intervene early.

However, based on findings from the aforementioned European survey of rheumatology centres [11•], the use of assessment tools in routine transitional care practice does not seem particularly well embedded (with only 36.4% of surveyed services reporting the use of any checklist format tool as part of an individualised transition plan, slightly more so in paediatrics than adults). In addition, after examining the components of the tools reported as used by centres, the study found significant under-representation of key topics (including vocational readiness, mobility, living independently, travel and knowledge about the health system), thus raising the question as to how developmentally appropriate the tools were.

### Transition Readiness

Several recent reviews of transition readiness tools [45–47] have identified and examined a number of both condition-specific and non-condition-specific transition readiness tools, including the rheumatology-specific Readiness for Adult Care in Rheumatology (RACER) questionnaire [48]. However, the psychometric properties of this and other available transition readiness tools are limited or untested [45].

One non-condition-specific tool, the Transition Readiness Assessment Questionnaire (TRAQ) [49, 50], developed with a population of older adolescents (16–26 years), was evaluated by Zhang et al. [45] as the most robustly validated transition readiness tool to date. However, a psychometric evaluation of the TRAQ in a younger adolescent population (mean age 15.3 years) was unable to validate the measure [51], raising questions about whether the TRAQ is a suitable measure to evaluate readiness among younger adolescents. Other concerns with this tool have been raised in relation to its predominantly medically focused orientation, which does not address all aspects of the transition process, prompting the development and psychometric evaluation of more holistic tools such as the revised ON TRAC questionnaire [52].

Another aspect that has become increasingly apparent from recent examinations of available transition readiness tools is the significant variation in conceptualisations of transition readiness and the resulting challenges for measurement development and validation [47]. Therefore, both clinicians and researchers should pay careful attention to what it is that is really being measured. For example, one could argue that most available transition readiness tools are in fact transfer (rather than transition) readiness measures. Another example of such conceptual variation can be found in the multifaceted nature of the readiness judgement. Most transition readiness measures should be interpreted as the AYA’s perception of their transition readiness whilst others, acknowledging the role and impact of parents and parental readiness on health transition [21, 23], also incorporate parent-reported versions of the measures—with few of the non-condition-specific tools [45, 53] and more of the condition-specific tools [54–56] incorporating both AYA- and parent-reported readiness.

In addition, there are limitations of these measures such as their self-reported nature or the lack of assessment of actual mastery of skills. For example, a study of AYA with liver transplants found that young adults (> 18 years) had significantly greater self-reported healthcare self-management compared with younger adolescents, but less than half of the young adults consistently managed their healthcare independently, made their own appointments or understood health insurance issues [56]. Similarly, a study of older adolescents with liver transplants found that those reporting greater perceived self-management were associated with being at greater risk for medication non-adherence [57]. Therefore, it is paramount to recognise that such skills do increase with age [58, 59] and bear these trajectories in mind when using such assessment tools and use them in conjunction with routine developmental clinical assessment.

There are many challenges of assessing transition readiness in the clinic setting in addition to those of assessing mastery of skills. These include the complexity of conditions, the competing agendas (of various professionals, the parent(s) and the young person), limited time, limited personnel and resources, multiple other questionnaires, context of relapsing disease and the potential for regression of readiness. When studying transition readiness, these factors and their impact on any assessment should be considered. An attempt to simplify the practicalities of assessment concentrating on ensuring routine psychosocial screening could be just as effective, with the assistance of tools such as the HEEADSSS [60] and the addition of trigger questions for health transition specific skills, medicine management and adherence with appropriate documentation—e.g. THRxEADS [61].

It is also interesting to consider how AYA with LTHC compare with their healthy peers in their health knowledge and skills. In a study of 494 young people with and without LTHC (mean age 19.3 years), Eaton et al. [62] reported that AYA with LTHC had greater transition readiness and self-involvement in completing medical tasks with less parent involvement than their healthy peers, but there was no difference in general self-efficacy and the ‘managing daily activities’ subscale of the TRAQ. These findings support the idea that understanding ‘normal’ AYA development is key to understanding the impact of a LTHC during adolescence and young adulthood.

It could also be argued that AYA are no different from older adults in their health-related skills. For example, Fishman et al. [63] conducted a survey with 141 adults with IBD (aged 25–55 years) and found that 37% could not recall drug doses, 35% could not recall drug frequency and 73% of those on a biologic did not cite infection as a side effect [63]. This suggests that perhaps by investing more resources during adolescence and young adulthood (as the life stage when both risky and health-promoting behaviours become established), adult health could also improve.

### Experience and Satisfaction

Patient experience measures for transition are also available. These include the Adolescent Assessment of Preparation for Transition (ADAPT) [64] for AYA aged 16–17 years, and the Mind the Gap scale [65] which measures satisfaction with transitional care for both AYA and parents. The novelty of the Mind the Gap scale, which was first developed as part of a multicentre study of transitional care in UK rheumatology [24], is that it measures the gap between what the individual considers the best practice and what they are currently experiencing [65] and therefore is potentially more informative.

Patient experience measures specifically addressing the event of transfer and self-efficacy have also been developed, such as the On Your Own Feet Transfer Experience Scale (OYOF-TES) [39] and the On Your Own Feet Self-Efficacy Scale (OYOF-SES) [40], both of which used successfully in the recent evaluation of a clinical transition pathway for adolescents with juvenile-onset RMD in the Netherlands [38] (Table 1).

Successful engagement of AYA has been reported to require a team-based approach [66], and changes in the team climate have been identified as predictors of the quality of transitional care delivery [67]. Therefore, attention to how such teams communicate and deliver transitional care for AYA over time is important. Checklists for professionals have been developed to support this. In the UK, a team-planning template was developed as part of the first UK rheumatology transition research programme [33] to coordinate team-working with AYA during the transition process [68]. This work was later on adopted by others in the UK and used across hospitals and specialties [34]. Internationally, Akre and colleagues developed a checklist for rheumatology professionals from a consensus exercise among an international expert panel [69].

Finally, a note of caution when using any readiness, knowledge, skills, experience or satisfaction measures, it is fundamental that rheumatology team members have the knowledge and skills to address any issues raised by AYA and/or their parents. As noted above, studies in both paediatric [13, 14] and adult settings [15] continue to report unmet needs in this area, including transitional care. Therefore, a key component of transitional care is ensuring staff training in AYA-specific health issues, an aspect which is also recognised in EULAR guidance [8••].

## Transition Outcomes

There is increasing evidence to support the benefits of effective transitional care, both in rheumatology (see [35] and Table 1) and beyond: Fenton et al. [70] reported that increased transition readiness in adolescents with chronic kidney disease was associated with positive outcomes in terms of reduction in the number of visits to the emergency room and improved adherence to medication. A large transition research programme in the UK involving 27 healthcare organisations and 374 AYA [71••] considered what components of service provision predicted better transition outcomes and found that promotion of health self-efficacy predicted satisfaction, appropriate parental involvement predicted well-being and meeting the adult team before transfer predicted improvements in participation and autonomy in appointments [72].

As yet, there is still no gold standard outcome measure for transition [73] and, arguably, some glaring omissions can be identified in the lists of proposed outcome measures to date [73, 74]. For example, vocational outcomes and psychological outcomes are less frequently proposed, with a bias towards more traditionally medical health outcomes (such as disease and medication knowledge) as well as a bias towards assessing knowledge (rather than skills).

Moreover, there is one key challenge underlying transition outcome measurement, which is how to define successful transition, given that such definition will be contingent upon the perspective(s) being considered. For example, from a health professional perspective, continuity of care during transition is critical; consequently, confirmation of engagement with the adult services will be an important and measurable outcome of transition from this perspective [75]. Considering this outcome measure, a large study of 1623 patients attending a single institution found that clinics with higher proportions of successfully transferred patients had a lower median number of days between the last paediatric visit and first adult visit and higher transitional care quality scores [76].

## System-Level Transition Strategies

The World Health Organization recently called for a shift from a focus on youth-friendly health services to consideration of adolescent-responsive health systems [77]. In keeping with this call, we need to consider the systems wherein our paediatric and adult rheumatology services sit and whether or not they too promote positive youth development, provide developmentally appropriate healthcare (DAH) and proactively nurture the mastery of transitional skills. For example, a particular rheumatology clinic may routinely promote autonomy for AYA with SLE, but if those same AYA are not given similar opportunities when they attend the renal clinic, they will be left with mixed messages and at risk of disengagement from services. There is an extensive literature on youth friendliness of health service provision from the perspective of AYA [78], but much less is known about how youth-friendly and developmentally appropriate are the institutions (or indeed the systems) within which such services are delivered.

An ethnographic study of 3 UK hospitals (including adult and paediatric rheumatology services) found significant variation and inconsistencies in how different health professionals and hospital managers across specialities and organisations understood and perceived the concept of DAH for AYA [79]. Furthermore, diverse values and commitment towards the care of AYA and provision of DAH were observed, leading to inequities in skills and experience. There is a need for organisation-wide strategies to ensure implementation of DAH (including transition) across systems. The aforementioned ethnographic study of UK hospitals proposed a toolkit for health professionals to support the implementation of DAH for AYA in hospitals [80]. This toolkit is equally relevant to rheumatology as it is to any specialty providing care for AYA. There is also a rheumatology-specific toolkit to evaluate the developmentally appropriateness of AYA rheumatology services [81].

Transitional care, by definition, is concerned with both child and adult rheumatology services. However, organisational and policy gaps around transition services are common and the integrated planning and commissioning of such services is often lacking [82]. UK-based research reported that service commissioners perceived a lack of national and local policy to guide integrated commissioning (i.e. joint institutional arrangements to commission services across health and social care) and found that differences in organisational culture, funding arrangements and work practices made inter- and intra-agency coordination and cross-boundary continuity of care difficult to achieve [82]. This has been highlighted in rheumatology with respect to the continuity of funding for biologics across the transfer period and, for this reason, specifically included in the EULAR guidance [8••].

There are many challenges at a system level for health transition both within and between systems, namely the challenges of coordination, communication, consistency, consensus and continuity. This is particularly relevant for AYA with multisystem RMD, such as SLE or vasculitis, who will require health transition coordinated in several specialty clinics and would particularly benefit from system-level transition strategies. Hepburn et al. [83] examined the policy profile of paediatric-to-adult care transitions across 9 jurisdictions in high-income countries, reporting the need for jurisdictions to provide flexibility and funding, enable cross-sectoral collaboration, including communication and coordination, and recommended that healthcare providers engage health system planners in the design and evaluation of system-level, policy-sensitive transition strategies [83].

## Conclusions: Potential Ways Forward for Transition Research in Rheumatology

Several authors have reflected on why it is taking so long to embed transitional care into rheumatology and other specialties. One argument is that we have failed to consider the context of AYA development [9, 84]. We would therefore propose that rheumatology moves from transition being the primary focus for adolescent rheumatology to adopting a life course approach to rheumatology, considering instead adolescent and young adult rheumatology [85] to bridge the gap between the disciplines, as colleagues in oncology have done for a long time [86]. AYA rheumatology might be better placed to successfully address not only the health transition of AYA with RMD but also all the other key transitions of this life stage (e.g. pubertal, educational and social) as AYA grow up, regardless of the setting of services.

Throughout this paper, a number of areas for further research have been highlighted. Future transition research should bear in mind not only the life stage of adolescence and young adulthood but also the social-ecological model of transition readiness, which focuses on the interactions between AYA, parents/caregivers and providers (and the systems they are part of) as these are the likely targets of any intervention to improve health transitions [87].

In 2007, there was a call to recognise emerging adulthood in rheumatology [88], and in the decade since, AYA rheumatology has gathered momentum alongside transitional care developments (see [35•] and Table 1). In the UK, the development of the Barbara Ansell National Network for Adolescent Rheumatology (http://bannar.org.uk) has been integral to progress this further, with membership from paediatric and adult rheumatology involved as well as a national youth advisory panel [89]. Hopefully, with increased collaboration between paediatric and adult rheumatology and active involvement of AYA and their families, we will eventually get health transition right and developmentally appropriate for everyone involved.
